# SirT1—A Sensor for Monitoring Self-Renewal and Aging Process in Retinal Stem Cells

**DOI:** 10.3390/s100606172

**Published:** 2010-06-21

**Authors:** Chi-Hsien Peng, Yuh-Lih Chang, Chung-Lan Kao, Ling-Ming Tseng, Chih-Chia Wu, Yu-Chih Chen, Ching-Yao Tsai, Lin-Chung Woung, Jorn-Hon Liu, Shih-Hwa Chiou, Shih-Jen Chen

**Affiliations:** 1 Department of Ophthalmology, Shin Kong Wu Ho-Su Memorial Hospital and Fu-Jen Catholic University, Taipei 11101, Taiwan; E-Mail: chpeng1008@gmail.com (C.-H.P.); 2 Department of Medical Research and Education, Taipei Veterans General Hospital, 201 Shih-Pai, Road, Section 2, Taipei 11217, Taiwan; E-Mails: ylchang@vhgtpe.gov.tw (Y.-L.C.); ycchen@vghtpe.gov.tw (Y.-C.C.);; 3 Department of Ophthalmology, Taipei Veterans General Hospital, 201Shih-Pai, Road, Section 2, Taipei 11217, Taiwan; E-Mail: ccwu@vghtpe.gov.tw (C.-C.W); 4 Department of Physical Medicine & Rehabilitation and Surgery, Taipei Veterans General Hospital, 201Shih-Pai, Road, Section 2, Taipei 11217, Taiwan; E-Mails: clkao@vghtpe.gov.tw (C.-L.K); lmtseng@vghtpe.gov.tw (L.-M.T); 5 Institute of Pharmacology, National Yang-Ming University, No. 155, Sec 2, Linong Street, Taipei 11221, Taiwan; 6 Institute of Clinical Medicine, National Yang-Ming University, No. 155, Sec 2, Linong Street, Taipei 11221, Taiwan; E-Mails: DAC58@tpech.gov.tw (C.-Y.T.); Dac91@tpech.gov.tw (L.-C.W.); 7 Department of Ophthalmology, Taipei City Hospital, Taipei, Taiwan; 8 Department of Ophthalmology, Cheng Hsin General Hospital, Taiwan; E-Mail:jhliu@chgh.org.tw (J.-H.L)

**Keywords:** retinal stem cells, aging, SirT1, resveratrol

## Abstract

Retinal stem cells bear potency of proliferation, self-renewal, and differentiation into many retinal cells. Utilizing appropriate sensors one can effectively detect the self-renewal and aging process abilities. Silencing information regulator (SirT1), a member of the sirtuin family, is a NAD-dependent histone deacetylase and an essential mediator for longevity in normal cells by calorie restriction. We firstly investigate the SirT1 mRNA expression in retinal stem cells from rats and 19 human eyes of different ages. Results revealed that SirT1 expression was significantly decreased in *in vivo* aged eyes, associated with poor self-renewal abilities. Additionally, SirT1 mRNA levels were dose-dependently increased in resveratrol- treated retinal stem cells. The expression of SirT1 on oxidative stress-induced damage was significantly decreased, negatively correlated with the level of intracellular reactive oxygen species production. Treatment with resveratrol could effectively further reduce oxidative stress induced by H_2_O_2_ treatment in retinal stem cells. Importantly, the anti-oxidant effects of resveratrol in H_2_O_2_-treated retinal stem cells were significantly abolished by knockdown of SirT1 expression (sh-SirT1). SirT1 expression provides a feasible sensor in assessing self-renewal and aging process in retinal stem cells. Resveratrol can prevent reactive oxygen species-induced damages via increased retinal SirT1 expression.

## 1. Introduction

The silent information regulator 2 (Sir2), a NAD^+^-dependent protein deacetylase, promotes longevity in yeast and mammalian cells [1]. Cells with an increase in Sir2 activity display a much longer lifespan than wild-type cells. Sir2 has an effect on anti-aging, and cells lacking Sir2 have a reduced replicative lifespan. This event may be regulated through hypersilencing of the formation of extrachromosomal ribosomal DNA circles in the nucleoli; a known cause of senescence in yeast [1]. Caloric restriction (CR) has already been reported as the key contributor to longevity in species from yeast and nematodes to rodents and monkeys. It is thought that CR slows the metabolic rate, generating more free NAD co-factors, resulting in greater active Sir2 deacetylase activity, and an extended lifespan [2,3]. Thus, The NAD^+^ dependency links Sir2 activity to metabolic changes. Furthermore, Sir2 and other related members of the sirtuin family are highly conserved from yeast to mammalian cells. The SirT1 gene encodes a member of the sirtuin family of proteins, homologs of the Sir2 gene in yeast, and contains the catalytic core domain of Sir2 [4,5]. SirT1, which resides in the nucleus, binds and deacetylates p53, NF-κB, and forkhead transcription factors, as well as histones [6]. SirT1 has many biological functions, including transcription regulation, cell differentiation inhibition, cell cycle regulation, and anti-apoptosis. In addition, SirT1 is postulated to have capacity of neuroprotection and prevents from neurodegeneration and diabetes [7–9]. However, the role of SirT1 being a sensor in self-renewal and aging in mammals remains to be further evaluated.

Retinal degeneration such as age-related macular degeneration (AMD) is the leading cause of irreversible blindness in developed countries and accounts for 8.7% of all cases of blindness globally [10]. A complex interaction of demographic, genetic and environmental risk factors is thought to exist in the etiology and pathogenesis of AMD. The most established risk factors associated with AMD are advanced age, cigarette smoking, diet and race [11]. Genetic variations in complement activities (complement factor H, component 2 and factor B) and in mitochondrial-associated protein (age-related maculopathy susceptibility 2) lead to excess macular inflammation and retinal oxidative stress involved in the development of AMD [12]. Persistent chronic inflammatory and oxidative cell damage gradually results in permanent photoreceptor loss and retinal pigment epithelium dysfunction in patients with advanced AMD. Replacement of lost retinal cells by stem cell-based therapy offers a potential therapeutic approach to restore visions in advanced AMD patients. Retinal stem cells (RSCs), derived from pigmented ciliary margin of the eyes, have been isolated and defined as cells with the capacity of self-renewal and multilineage differentiation [13]. These cells bear multipotency of proliferation, self-renewal and differentiation into bipolar neurons, Muller glia and photoreceptors. Murine RSCs transplanted into damaged or dystrophic retina are shown to differentiate into variable retinal neurons [14–16]. RSCs possess the potential to develop the transplantation strategies for retinogenesis in retinodegenerative diseases [17,18].

The specific aim of this study was to evaluate the effects of SirT1 activation on self-renewal and oxidative stress-induced aging of RSCs. SirT1 mRNA expression levels were examined in RSCs from rats and human eyes of different ages. Resveratrol (trans-3,5,4′-trihydroxystilbene, RV) is found in the skin of red grapes, nuts, pomegranates, and red wine. It is a SirT1-activating compound and possesses the ability to scavenge free radicals with promising neuroprotective effects [19–24]. To investigate the role of RV in the self-renewal and aging of RSCs, H_2_O_2_ was used to mimic the effect of oxidative stress in RSCs to explore how RV and SirT1 protect the retinal neurons from oxidative stress.

## 2. Results and Discussion

### 2.1. Proliferation, Differentiation and Gene Expression of Retinal Stem Cells

**Figure 1.**
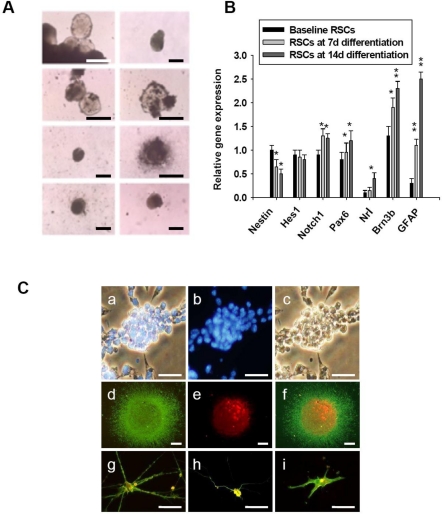
Isolation of rat RSCs. (A) *In vitro* culture of rat RSCs. The floating neurospheres were derived from RSCs and cultivated in serum-free medium with EGF and bFGF. Bars: 100 μm. (B) Comparison of the gene expression among fresh RSCs at baseline, RSC after 7 and 14 day differentiation *in vitro* culture. The symbol * indicated a significant difference among RSC groups at baseline, day 7 and day 14 differentiation (*p < 0.05; **p < 0.01). Data shown here are the means ± SD of three independent experiments. (C) The differentiation ability of RSCs (a-c) RSCs aggregated into spheroid body (a and b, blue: DAPI) and expressed nestin protein (d and f; Nestin: green color) and stained with musashi1 (e and f; Musashi1: red color). (f) The merged image of nestin and musashi1 markers was co-localized in RSCs. (g–i) After 14 days differentiation, the retinal differentiation of RSCs was evidenced by morphology and immunofluorescein study. Fluorescent images were illustrating the differentiated RSCs and positively immunoreacted for GFAP (g; GFAP: green color; BrdU: red color), Thy-1 (h; Thy-1: green color; BrdU: red color), and rhodopsin (i; rhodopsin: green color; BrdU: red color). Bar: 100 μm.

For *in vitro* culture of RSCs, we harvested the freshly isolated RSCs from eight SD rats. The harvesting and culture of RSCs was done as previously described [25]. In the serum-free culture, RSCs began expansion and formed neurospheres (Figure 1A). The number of neurospheres generated from RSCs after seven days of culture was 17.1 ± 0.6 spheres per 5,000 viable cells. During the process of RSC differentiation, the expressions of seven major genes were evaluated at molecular level by RT-PCR (Figure 1B). The expression level of nestin was defined as 1, and Table 1 showed the primer pairs used in the experiment. *Nestin* is a maker of neural progenitor cells. *Hes1* and *Notch1* inhibit neural differentiation and maintain progenitor cells, and both genes are also play a role in late RSCs to generate glia. *Pax6* appears a key controller for the development of eyes and other sensory organs. Lack of *Pax6* expression could induce expression of ectopic eyes, and produce a wide spectrum of ocular defects such as aniridia in humans with heterozygous mutants [26]. *Nrl*, *Brn3b*, and *GFAP* were involved in the development of the rod photoreceptors, retinal ganglion cells, and glial cells, respectively. During the process of retinal differentiation, we compare the relative gene expressions at day 7 and 14 differentiation with those at baseline. The results showed that the expression of *Nestin* was significantly decreased after 7 and 14 day differentiation (*p < 0.05). Both *Hes1* and *Notch1* were functional in Notch pathway, which involved in retinal development. Only *Notch1* showed significantly higher after differentiation (*p < 0.05). The expression of *Pax6* revealed higher in RSCs after differentiation than fresh RSCs at baseline (*p < 0.05). After 7 and 14 day differentiation, *Brn3b*, *Nrl* and *GFAP* also showed significantly higher expression (*p < 0.05). In general, the gene expression features appeared to be consistent with the process of generating the mature retina from progenitor cells [26,27].

Figure 1C shows the morphology and phenotype of RSC proliferation and differentiation. Initially, RSCs aggregated into spheroid formation (Figure 1Ca; merged; blue: DAPI), and both neural progenitor markers Nestin (green) and Musashi1 (red) were highly detected in these neurospheres by using immunofluorescent study (Figure 1Cf; merged). Without expression of mature retinal and neural marker, these neurospheres kept self-renewal and proliferation in an undifferentiated state. To investigate the capacity of retinal differentiation, these spheroid-like Nestin and Musashi1-positive RSCs were further cultured in the differentiated medium. Immunophenotypic analysis on day 14 showed that differentiated cells expressed GFAP-positive glial cells (Figure 1Cg), Thy-1-positive retinal ganglion cells (Figure 1Ch) and rhodopsin-positive photoreceptos (Figure 1Ci).

### 2.2. Detection of SirT1 mRNA and Telomerase Activity in Rat Retinal Stem Cells

**Figure 2.**
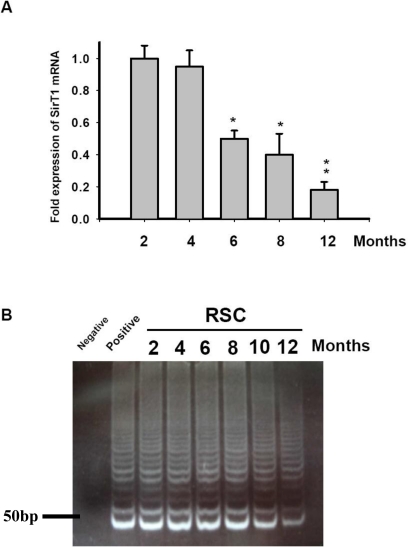
Detection of SirT1 mRNA and telomerase activity in rat RSCs.(A) The amount of SirT1 mRNA was detected by Q-PCR from RSCs of SD rats of different ages, and. *p < 0.05; **p < 0.01 *versus* 2- and 4-month-old RSC groups. Data shown here are the means ±SD of three independent experiments. (B) Telomerase activities were quantified using a telomerase repeat amplification protocol (TRAP) assay. RSCs from rats in different ages were lysed in CHAPS lysis buffer, and equal protein quantities (300 ng) were subjected to TRAP analysis to determine telomerase activity. Negative control: without cell lysate; positive control: H1299 cell lysate.

To investigate the relationship between aging and SirT1 gene expression, the SirT1 mRNA expression fold in RSCs was evaluated in SD rats of different ages (2, 4, 6, 8, and 12 months). The expression level of SirT1 in 2-month-old group was defined as 1. We used the *t*-test for comparing each group of different ages. Figure 2A shows that the SirT1 mRNA expression level in the RSCs of the 12-month-old groups were significantly lower than those of the 2-month-old (**p < 0.01) and 4-month-old (**p < 0.01) groups, as measured by qRT-PCR. The SirT1 mRNA expression level was also significantly decreased in the 6- and 8- month-old groups compared to the 2-month-old (*p < 0.05) and 4-month-old (*p < 0.05) groups. However, the SirT1 mRNA expression level of 2-month-old RSCs was not significantly different from that of 4-month-old cells (p > 0.05). The enzyme telomerase allowed for replacement of DNA known as telomeres, which were otherwise lost when a cell divided via mitosis. It was possible that the telomere erosion contributed to the normal aging process, and a variety of premature aging syndromes were associated with short telomeres [28,29]. Telomere shortening was associated with cellular senescence and the mean telomere length had emerged as a replicative clock and a biomarker for biological aging *in vivo*. In our experiments, telomerase activities in RSCs from different ages (2, 4, 6, 8, 10 and 12 months) of rats were detected using a telomerase repeat amplification protocol (TRAP) assay. H1299 cell lysate was designed as a positive control, and the negative control was without cell lysate. Telomerase activities were found to be almost constant among RSC of 2-, 4-, 6-, 8-, and 10-month-old groups, but slightly decreased in the 12-month-old group (Figure 2B). The result revealed that RSCs from the older rats yielded lower levels of telomerase.

### 2.3. Detection of SirT1 mRNA in Human Retinal Stem Cells

Next, to study the connection between SirT1 and aging in human RSCs, the expression levels of SirT1 mRNA in RSCs isolated from the posterior ciliary margin of human donors with different age were examined by Q-PCR. Table 2 lists the characteristics of the patients for RSC analysis.

**Figure 3.**
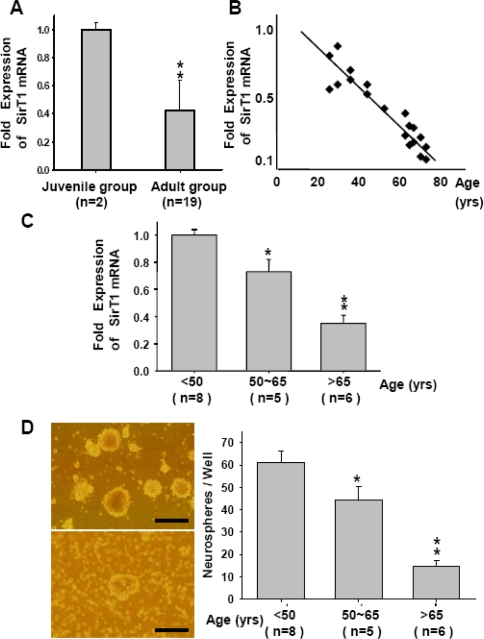
Detection of SirT1 mRNA in RSC isolated from the posterior ciliary margin of human donor. (A) The SirT1 mRNA expression level detected by qPCR in RSCs from adult groups (n = 19) was significantly decreased compared to that in RSCs from juvenile group with donor age younger than 10 years old (n = 2, **p < 0.01). (B) The regression plot showed that the SirT1 level was negatively correlated with age in RSCs. (C) Human samples were divided into three groups based on age. The bar chart demonstrated that the SirT1 mRNA level was significantly decreased with age. (D) Neurospheres formation showed increased in patients younger than 50 years old (left upper) than those older than 65 years old (left lower). Bar: 100 μm. Data shown here are the mean ± SD of three experiments.

A total of 23 eyes from 12 subjects were included in this study. In juvenile group, four eyes were from two subjects younger than 10-years-old. The other 19 eyes from 10 subjects were from adults (mean age: 50.60 ± 17.93 years old, nine males and one female). Most donors were deceased due to traffic accidenta, stroke, or cancer. One subject had only a single donor eye due to exposure keratopathy-induced cornea edema, which would not be a good candidate for corneal transplant. The expression level of SirT1 in juvenile group was defined as 1. We used the *t*-test to comparing juvenile and adult groups. Figure 3A showed that the SirT1 mRNA expression level in RSCs from adult group (n = 19) was significantly decreased compared to that in RSCs from juvenile group (n = 2, **p < 0.01). Interestingly, the qRT-PCR analysis further demonstrated that the SirT1 mRNA expression levels were significantly decreased in an age-dependent matter. In the adult group, the results showed that there was a negative regression relationship between the age and SirT1 mRNA expression levels in RSCs (Figure 3B).

RSCs from the 19 eyes in the adult group were further divided into three groups based on ages. Group 1 had eight eyes with a donor age younger than 50 years old. Group 2 had five eyes with donor ages between 50 and 65 years old. The other six eyes with donor ages older than 65 years old were categorized in group 3. Next, we defined the level of SirT1 mRNA in group 1 as 1. Figure 3C showed that the SirT1 mRNA expression level in group 3 RSCs were significantly lower than group 1 RSCs (**p < 0.01), as measured by qRT-PCR. The SirT1 mRNA expression level was also significantly decreased in group 2 RSCs compared to group 1 RSCs (*p < 0.05). For self-renewal assay, it was also observed that the number of floating neurospheres on day 7 was significantly reduced in the group 3 compared with that in the group 1 (Figure 3D). Neurospheres formation showed obviously decreased in group 3 (Figure 3D, left lower) than in group 1 (Figure 3D, left upper). The mean number of neurospheres in group 1 was 61.3 ± 4.8 spheres per well, which was significantly higher than group 2 of 45.6 ± 5.1 spheres per well (*p < 0.05). The number of neurospheres in group 1 also showed significantly higher than group 3 of 16.1 ± 2.1 spheres per well (**p < 0.01). These data indicated that both SirT1 expression level and self-proliferation ability were decreased with age. SirT1 could be indicated as a sensor of self-renewal of human RSCs as well as of RSCs aging.

### 2.4. Expression Activities of SirT1 in RSCs of Different Aged (Month-old) Rats by SirT1 Promotor

**Figure 4.**
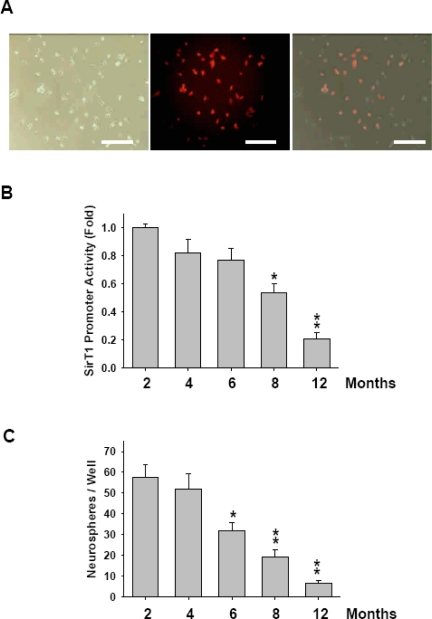
Expression activity of SirT1 in RSCs of different aged rats was evaluated by Sirt1 promotor. (A) RSCs from 2-month-old rats were transfected with pDsRed-SirT1p. Left: bright light. Middle: RFP. Right: merge. (Bar: 40 μm). (B) pGL2-SirT1p was transfected into rat RSCs. After harvesting the cells, lysates were assayed for luciferase activity. All relative luciferase activities were normalized with control Renilla luciferase expression. *p < 0.05; **p < 0.01 *versus* 2- and 4-month-old groups. (C) After 7 days of serum-free medium culture, the number of neurospheres was counted. Data shown here are the mean ±SD of three experiments. *p < 0.05; **p < 0.01 *versus* 2- and 4-month-old groups.

Since SirT1 level was highly associated with age, we set up an aging-indicator system using *SirT1* promoter. pDsRed-SirT1p, where the CMV promoter was replaced by *SirT1* promoter, was transfected into rat RSCs and observed by fluorescence microscopy. Presence of RFP-positive cells demonstrated that the transcriptional mechanism, which drove the expression of SirT1, simultaneously turned on the expression of RFP (Figure 4A). An alternative monitor method was to transfect pGL2-SirT1p into RSC derived from 2- to 12-month-old rats. Figure 4B showed that the activity of SirT1 promoter in the RSCs of the 12-month-old groups were significantly lower than those of the 2- and 4-month-old groups (**p < 0.01). The activity of SirT1 promoter was also significantly decreased in the 8-month-old group compared to the 2- and 4-month-old groups (*p < 0.05). Luciferase assay revealed that the activity of SirT1 promoter was repressed associated with age (Figure 4B), which was consistent with the expression level of SirT1 mRNA (Figure 2A). Figure 4C showed the mean number of neurospheres in 8- and 12-month-old groups was significantly lower than 2- and 4-month-old groups (**p < 0.01). The number of neurospheres in 6-month-old group also showed significantly lower than 2- and 4-month-old groups (*p < 0.05). Thus, the above data suggested that SirT1 could be a sensor of cellular activity state.

### 2.5. Resveratrol (RV) Increase of Cell Viability in Retinal Stem Cells via the Activation of SirT1

**Figure 5.**
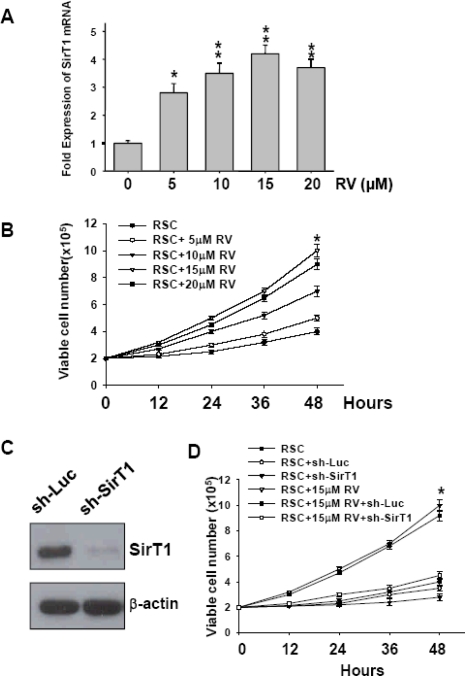
Resveratrol (RV) increase cell viability in RSCs via the activation of SirT1. (A) RSCs from 12-month-old rats were treated with 0, 5, 10, 15, and 20 μM of RV for 48 hours. *p < 0.05: 5 μM RV *versus* control. **p < 0.01: 10, 15 and 20 μM RV *versus* control. (B) Cell viability for 48 hours were shown in RSCs treated with 0, 5, 10, 15, and 20 μM of RV. (*p < 0.05: 15 μM RV *versus* control). (C) Western blots showed SirT1 expression levels in RSCs transduced with lentivirus carrying luciferase shRNA (sh-Luc; vector control), and SirT1 shRNA (sh-SirT1). (D) Cell viability for 48 hours were shown in RSCs treated with 0 or 15 μM of RV after knock down by sh-Luc or sh-SirT1 (*p < 0.05: 15 μM RV *versus* control; *p < 0.05: 15 μM RV *versus* 15 μM RV+ sh-SirT1). Data shown here are the mean ±SD of three experiments.

Recently, RV has been proven as a SirT1 activator. To further clarify our findings and to explore the effect of RV on RSCs under oxidative stress, we want to determine the most optimal concentration of RV based on the SirT1 expression and cell viability. Firstly, RSCs derived from 12-month-old rat were treated with different doses of RV (0, 5, 10, 15, and 20 μM). Using Q-PCR, the SirT1 expression level was evaluated, and the results suggested that 15 μM RV was the optimal concentration to stimulate the expression of SirT1 mRNA in RSCs (**p < 0.01, Figure 5A). We further investigated the cell viability, and the results also showed that the most viable cell number was observed after 15 μM RV treatment for 48 hours (*p < 0.05, Figure 5B). For evaluation the role of SirT1 in RV-treated RSCs, the SirT1 gene was silenced using a small interfering RNA (sh-SirT1) expressed from a lentiviral vector. Western blots showed SirT1 expression levels in RSCs transduced with lentivirus carrying luciferase shRNA (sh-Luc; vector control), and SirT1 shRNA (sh-SirT1) in figure 5C. The growth curve revealed that downregulation of SirT1 reduced RV-induced proliferation rate in RSCs (*p < 0.05, Figure 5D).

### 2.6. Resveratrol Protects Against H_2_O_2_-induced Oxidative Stress in RSCs via the Activation of SirT1

**Figure 6.**
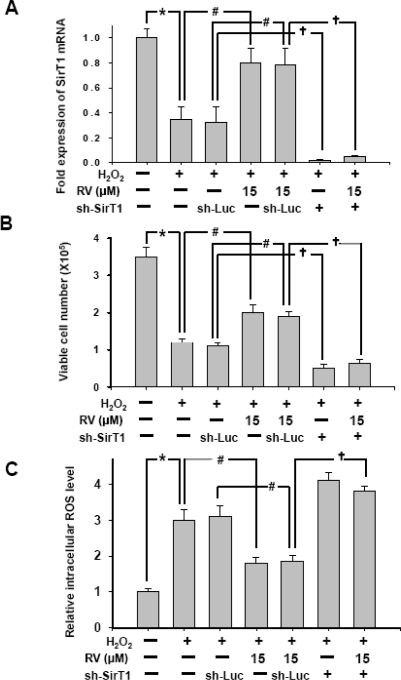
Resveratrol Protects Against H_2_O_2_-induced damage in RSCs via the Activation of SirT1. (A) Total RNA was extracted, and SirT1 mRNA levels were examined by Q-PCR. After 2 days of treatment with 15μM of RV, rat RSC were treated with 100 μmol·L^−1^ H_2_O_2_ or vehicle for 8 h, followed by detection of SirT1 mRNA level (A, fold of control) (B) Cell viability for 48 hours were shown in RSCs treated with 0, 15 μM of RV after knock down by sh-Luc or sh-SirT1 (C) Reactive oxygen species (ROS) production was detected (C, fold of control). *p < 0.05. #p < 0.05. **†**p < 0.05. Data shown here are the mean ±SD of three experiments.

In this experiment, H_2_O_2_ was used to mimic oxidative stress-induced damage in RSCs. After 2 days of treatment with or without 15 μM RV, RSCs were treated with 100 μmol·L^−1^ H_2_O_2_ or vehicle for 8 h, followed by the detection of SirT1 mRNA level. We defined the SirT1 mRNA level in control group as 1. For evaluation the protective effect of RV against H_2_O_2_ -induced oxidative stress and the role of SirT1, these RSCs were treated with 0 or 15 μM of RV following knock down by vector sh-Luc or sh-SirT1. This experiment was divided into seven groups for detection of the expression of SirT1, cell viability and the level of intracellular ROS. H_2_O_2_ treatment dramatically decreased the expression of SirT1 and cell viability (*p < 0.05, figure 6A, 6B), however, the addition of RV could efficiently recover the expression of SirT1 mRNA and the number of viable cells in H_2_O_2_-treated RSCs (#p < 0.05, figure 6A, 6B). We found that treatment of 15 μM RV significantly increased the expression of SirT1 mRNA and cell viability than those treated with H_2_O_2_ (#p < 0.05, figure 6A, 6B). In H_2_O_2_-treated RSCs, the protective effect of RV was significantly suppressed by sh-SirT1. The expression of SirT1 mRNA and the number of viable cells showed significantly decreased in RSCs treated with H_2_O_2_, 15 μM RV and sh-SirT1 compared to those treated with H_2_O_2_, 15 μM RV and sh-Luc (**†**p < 0.05, figure 6A, 6B).

Furthermore, the role of SirT1 in production of intracellular reactive oxygen species (ROS) was also examined. The amount of ROS was significantly increased in H_2_O_2_-treated RSCs (*p < 0.05, Figure 6C). The production of ROS was significantly suppressed by 15 μM RV in H_2_O_2_-treated RSCs (#p < 0.05, figure 6C). The anti-oxidative stress effect of RV was significantly suppressed by sh-SirT1 in H_2_O_2_-treated RSCs. The level of ROS showed significantly higher in RSCs treated with H_2_O_2_, 15 μM RV and sh-SirT1 than those treated with H_2_O_2_, 15 μM RV and vector sh-Luc (**^†^**p < 0.05, figure 6C). These data suggest that RV is a potential agent to rescue oxidative stress-induced damage in treated RSC cells. The effect of RV for anti-oxidative stress and decrease ROS production could be via the SirT1 pathway.

### 2.7. Resveratrol Promoted SirT1 Activity and Enhanced the Self-Renewal Ability to Increase the Numbers of Neurospheres in Aged-Retinal Stem Cell

**Figure 7.**
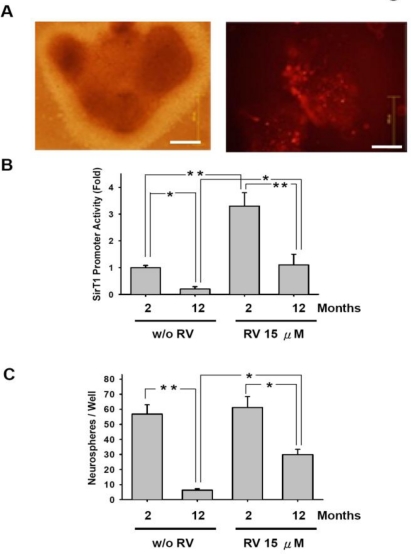
Resveratrol promoted SirT1 activities and enhanced the self-renewal abilities to increase the number of neurospheres in retinal stem cell. (A) RSCs isolated from 2- and 12-month-old rats were transfected with pDsRed-SirT1p. After 7 days of serum-free culture, RFP-positive cells were detected in spheres. (Bar: 40 μm) (B & C) RSCs isolated from 2- and 12-month-old rats were transfected with pGL2-SirT1p. After 7 days of serum-free culture with or without RV, cell lysates were assayed for luciferase activity (B) relative to 2-month-old rat RSC without RV treatment) and the number of neurospheres was counted; (C). *p < 0.05; **p < 0.01. Data shown here are the means ±SD of three independent experiments.

To directly monitor that whether RV regulated the SirT1 promoter activity in RSCs, cells from 2- and 12-month-old rats were transfected with either pDsRed-SirT1p or pGL2-SirT1p, and cultured in serum free medium with or without RV. RFP-positive cells were observed in spheres (Figure 7A). RV was found to effectively promote the activity of SirT1 promoter (Figure 7B). Treatment of RV activated the expression of SirT1 in RSCs from 2- (**p < 0.01) and 12-month-old rats (*p < 0.05). In the group treated with 15 μM RV, RSCs from 2-month-old rats showed higher expression of SirT1 (**p < 0.01) as well as in the group without RV treatment (*p < 0.05). RV was also evaluated the ability of promoting sphere formation. The results showed that RV effectively enhanced the sphere formation in RSCs from 12-month-old rats (*p < 0.05), but not in RSCs from 2-month-old rats (p > 0.05, Figure 7C). These data suggested that RV activated the expression of SirT1 and significantly enhanced the self-renewal ability of RSCs, especially RSCs from older rats.

### 2.8. Discussion

SirT1, a NAD+ dependent histone deacetylase, regulates lifespan and has an effect on aging. Some studies have shown the relationship between the aging change, apoptosis, and inducing oxidative stress by inhibition of SirT1. In this study, we firstly showed the RSCs and gene expression after differentiation (Figure 1). In aging RSCs, the data showed that SirT1 expression was limited (Figure 2). In human RSCs, the relative expression levels of SirT1 mRNA and the formation of neurospheres in RSCs from group 3 donors older than 65 were significantly lower than those in younger groups (Figure 3). In rats, RSCs from 12-month-old rat also showed lower activities of SirT1 promoter as well as the decreased number of neurosphere formation (Figure 4). To further clarify our findings and explore the underlying mechanism, H_2_O_2_ was used to mimic the damage of oxidative stress in RSCs. These cells were treated with different doses (5, 10, 15, and 20 μM) of resveratrol (RV). Treatment with RV promoted the cell proliferation, and the effect showed decreased after inhibition of SirT1 pathway (Figure 5). With H_2_O_2_ treatment, the addition of RV could efficiently recover the SirT1 mRNA expression levels and cell viabilities. This effect of RV was significantly suppressed in the H_2_O_2_-treated RSCs by sh-SirT1 (Figures 6A, 6B). The results showed that ROS production was significantly increased in sh-SirT1-treated RSCs compared to a vector control group under the same oxidative conditions (Figure 6C). The knockdown of SirT1 by sh-SirT1 significantly reversed the anti-oxidative-stress effect of RV in H_2_O_2_-treated RSCs (Figures 6B and 6C). This effect of RV was demonstrated significant in RSCs from older rats (Figure 7). To the best of our knowledge, this is the first study to report that SirT1 may be involved in the abilities of self-renewal and aging in RSCs, and that it may regulate the cell damage in oxidative stress-treated RSCs.

Telomere dysfunction and ROS damage are implicated in age-related decline of stem cells [30]. SirT1 may have a role in telomere maintenance during stem cell aging because of its essential role in gene silencing [31]. SirT1 gene-deleted mice display retarded growth rate, reduced survival to adulthood, and significantly slower rate of bone mineralization compared to wild type mice [32], all of which can be potentially explained by accelerated stem cell aging and attrition. Moreover, SirT1 could be involved in stem cell aging by suppression of age-related ROS generation [30,33,34]. Up-regulation of mitochondrial SirT1/PGC-1α will results in ROS generation decrease by forcing mitochondrial reprogramming to generate ATP by **β**-oxidation of fatty acids instead of carbohydrate catabolism [31,33–35]. Han *et al*. demonstrated that ROS-induced cell apoptosis was regulated via SirT1 in embryonic stem cells by deacetylation of p53 [34]. Much more apoptotic cells were observed in the SirT1 gene-deleted cells than in wildtype cells when embryonic stem cells were treated with H_2_O_2_ [34]. They further found that SirT1 cause p53 localization to mitochondria for regulating self-renewal and limiting ROS toxicity in embryonic stem cells. Recent studies also showed that SirT1 plays a role in redox status of hematopoietic stem cells and neural progenitor cells [36,37]. SirT1 might influence mitochondrial function and redox status with hematopoietic stem cells aging via its regulation of PGC-1α together [36,38]. Therefore, SirT1 was involved in the metabolism of these stem cells by maintaining proper ROS levels, limiting ROS accumulation and promoting longer stem cell life. The linkage of redox status to SirT1 in RSC aging needs to be further elucidated.

RV is a compound found largely in the skins of red grapes, nuts, pomegranates, and red wine. It possesses the ability to scavenge oxidatively generated free radicals, and prevents tumor progression by blocking NF-κB expression [17–21]. RV also has promising effects of neuroprotection, slowing aging and delaying the onset of chronic diseases [19,22–24]. In retina, the involvement of SirT1 in DNA repair and in maintaining energy homeostasis indicates the anti-apoptotic, neuroprotective effect of SirT1 expression [39]. RV, an activator of the SirT1 gene, possesses the potential as a scavenger for oxidative-stress-induced free radicals and toxicities. In human retinal pigment epithelium cells, RV was evident of neuroprotective effects associated with reduction of oxidative damage on phagocytic functions [40]. In addition, the anti-oxidative damage effect of RV could prevent cellular and molecular ocular inflammatory response by redox-sensitive NF-κB activation [41]. Furthermore, these anti-oxidative and anti-senescence effects of RV showed clinically improvement in human visual functions [42]. This improvement was associated with the reversal of retinal lipofusin, a waste of retina metabolite [42]. This ability of self-renewal was still not clear whether RV acts directly or indirectly in RSCs through SirT1. In the present study, the data showed that RV not only activated SirT1 expression levels in H_2_O_2_-treated RSCs, but also resulted in recovery from oxidative stress-induced damage. Moreover, ROS played a critical role in limiting the lifespan of organisms, as well as initiating the process of cellular senescence [43]. Increasing levels of ROS were also shown to restrict the lifespan of retinal stem cells. Importantly, with RV treatment, knockdown of SirT1 in RSCs increased the ROS production and further enhanced the oxidative stress-induced cell damage (Figure 6). Indeed, RSCs lead to impairment of self-renewal functions by inactivation of SirT1. SirT1 expression provided a feasible sensor in assessing self-renewal and aging process in RSCs.

The present results suggest that RV is a potential candidate for preventing the oxidative stress-induced cell damage in RSCs. These data also show that RV can prevent ROS-induced aging and cell damage via increased retinal SirT1 expression. RV appears to be a potential anti-oxidant that may prevent or slow down the production of ROS. The mechanism of RV-mediated SirT1 activation in aging and self-renewal RSCs needs further study. Its potential utility in protection against retinal dysfunction should be further determined.

## 3. Experimental Section

### 3.1. Isolation of Retinal Stem Cells

All animals used were treated in accordance with Animal Care and Use Committee guidelines at Taipei Veterans General Hospital. Eight Sprague-Dawley rats of each different ages (2, 4, 6, 8, and 12 months) were examined and anesthetized with intraperitoneal Phenobarbital. Human eyes were from 19 donors as list in Table 2. The harvesting and culture of RSCs was done as our previously described [25]. Briefly, the anterior eye segment was removed under a dissecting microscope and the neural retinal tissue was separated from the pigmented epithelial layer. The neural retinal tissue incubated for 10 min at 37 °C in Hanks’ balanced salt solution containing 0.05% trypsin and then mechanically dissociated with Pasteur pipettes in a DMEM/F-12 medium (Gibco) at a ratio of 1:1. These dissociated cells were then centrifuged at 150 g for 5 min. Afterwards, the enzyme solution was removed, and viable cells were counted by trypan blue exclusion and plated as 5,000 cells/200 μL per well in 96-well plates (Corning, Acton, MA, USA) replaced with serum-free culture media composed of DMEM/F-12 medium (Gibcol) with insulin (25 μg/mL), transferrin (100 μg/mL), progesterone (20 nM), putrescine (60 μM), sodium selenite (30 nM), and human recombinant EGF 20 ng/mL and bFGF 20 ng/mL (R&D Systems, Minneapolis, MN, USA).

The number of primary spheres generated in each well was assessed 7 days after plating. The primary suspended spheres were mechanically dissociated into single cells by trituration, and an aliquot was counted to determine the total number of cells. For secondary cultures, 500 cells/200 μL per well were plated in each well in 96-well plates using the same culture conditions. The number of secondary spheres in each well in each culture condition was scored 7 days after plating. In this state, we investigate the self-renewal abilities of RSCs.

The primary and secondary isolated spheres were plated on poly-L-ornithine-coated (15 μg/mL) glass coverslips in individual wells of 24-well plates (1.0 mL/well) in DMEM/F-12 medium containing 2% fetal calf serum (FCS; Gibcol) and withdrawing EGF and bFGF. Coverslips were processed 8 days later and fixed with 4% paraformaldehyde for 20 minutes for the observation of differentiation.

### 3.2 Quantitative Real-time RT-PCR

PCR reactions were performed on the ABI PRISM^®^ 7900HT Sequence Detection System and the ABI Prism 5700 SDS (Applied Biosystems). The amplification mixtures (50 μL) contained template DNA 1 μL, 2 X SYBR Green I Master Mix 25 μL, 0.4 μL of each primer (final concentration 200 nM), and DD water to volume. Copy number changes per haploid genome were calculated using the formula 2*^(Nt–Nline)–(Tt–Tline)^*, where *Nt* is the threshold cycle number observed for an experimental primer in the normal DNA sample, *Nline* is the threshold cycle number observed for a Line-1 primer in the normal DNA sample, *Tt* is the average threshold cycle number observed for the experimental primer in the test DNA sample, and *Tline* is the average threshold cycle number observed for a Line-1 primer in the test DNA sample. The conditions for amplification were as follows: one cycle of 94 °C for 2 minutes, followed by 50 cycles of 94 °C for 20 seconds, 57 °C for 20 seconds, and 70 °C for 20 seconds. Reactions for each primer set were performed in triplicate, and the threshold cycle numbers were averaged.

### 3.3. TRAP Assay

Telomerase activity was measured with the modified telomere repeat amplification protocol (TRAP) assay [31]. Pelleted cells were lysed with 100 μL of 1X CHAPS lysis buffer (10 mM Tris-HCl (pH 7.5), 1 mM EGTA, 0.5% CHAPS, 10% [v/v] glycerol, 5 mM β-mercaptoethanol, and 0.1 mM phenylmethylsulfonyl fluoride), incubated on ice for 30 min and centrifuged (13,000g, 4 °C, 30 minutes). Supernatant extracts were quantified for protein with a BSA Protein Assay Kit (Pierce, IL). TRAP assay was performed as previously described [44] with only minor modifications, with a set of primers (TS, 5′-AATCCGTCGAGCAGAGTT-3′; ACX, 5′-GCGCGGCTTACCCTTACCCTTACCCTAACC-3′; NT, 5′-ATCGCTTCTCGGCC TTTT-3′), and an internal standard, TSNT (5′-AATCCGTCG AGCAGAGTTAAAAGGCCG AGAAGCGAT-3′) [45]. Reaction mixtures were incubated (25 °C, 30 min) for telomerase-mediated extension and the samples were heated to 85 °C (10 minutes). Taq polymerase was added and each sample was amplified for 30 cycles of polymerase chain reaction (PCR) amplification (94 °C for 30 s, 59 °C for 30 s, and 72 °C for 90 s) in a DNA thermal cycler (GeneAmp PCR System 2400, PerkinElmer Co., Norwalk, CT, USA). TRAP products were resolved by 12.5% (w/v) non-denaturing polyacrylamide gel electrophoresis (PAGE) and visualized by staining with ethidium bromide. Relative telomerase activities were quantified by comparing signal intensities among lane and with the positive control (extract of H1299 cells) as 100%.

### 3.4. The Cloning of SirT1 Promotor, Transfection, and Dual-Luciferase Assay

For analysis of the *SirT1* promoter, a 2.8 kb fragment was PCR-amplified using mouse genomic DNA as a template, and subcloned into pDsRed2 (CMV promoter was replaced by *SirT1* promoter) and pGL2. The Lipofectamine 2000 system (Life Technologies, Grand Island, NY) was used for transfection of DNA into RSC cells. Briefly, 10^6^ cells/well were seeded onto 6-well plates 24 h before transfection. DNA solution (250 μl of OPTI-MEM containing 9 μg of pGL2-SirT1p reporter construct and 30 ng of pRL-tk Renilla normalizing luciferase vector) and reagent solution (5 μl of LF2000 and 250 μl of OPTI-MEM) were separately pre-incubated at 25 °C for 5 minutes. The transfections were then followed according to the manufacturer’s instructions. After 48 h of incubation, the cell lysates were prepared using the dualluciferase reporter assay system (Promega). Both firefly and Renilla luciferase activities were monitored by illuminator (TD20/20; Turner Designs, Sunnyvale, CA).

### 3.5. Knockdown of SirT1

The pLVRNAi vector was purchased from Biosettia Inc. (Biosettia, San Diego, CA, USA). The method of cloning the double-stranded shRNA sequence is described in the manufacturer’s protocol. The oligonucleotide 5′-AAAAGCAGATTAGTAGGCGGCTTGATTGGATCCAATCAAGCCGCCT ACTAATCTGC-3′ targeting human SirT1 (NM_012238, nt 1946–1966) were synthesized and cloned into pLVRNAi to generate Letiviral expression vector, pLVRNAi/sh-SirT1/1946. The lentiviral vector carryed luciferase shRNA is the vector control (sh-Luc; vector control). One day before transfection, plate 293FT cells (Invitrogen) at 5 × 10^6^ cells per 10 cm plate. Do transfection with Lipofectamine 2000 reagent (Invitrogen). Replace the medium with 5 mL of Opti-MEM I without serum. Prepare DNA-Lipofectamine 2000 complex with 2 μg of pVSVg, 4 μg of pCMV-ΔR8.91 and 10 μg of pLVRNAi/sh-SirT1/1946 in 3 mL of Opti-MEM I and add the complex to the plate. On the next day, replace the medium containing the complex with complete culture medium containing serum and antibiotics. Harvest the medium containing virus particles after 72 hour posttransfection. Spin down the medium to remove cell debris and save the supernatant. To determine the lentiviral titer, prepare 10-fold serial dilutions of viral stocks in complete culture medium ranging 1 to 10^6^. Remove culture media from the 293 FT cells and add 1 mL of the dilutions directly to the cells. If desired, add polybrene to each well to a final concentration of 8 μg/mL, and incubate at 37 °C overnight. The following day, replace the media containing virus with fresh complete culture media. After 3–4 days of transduction, observe GFP expression under fluorescent microscope and count GFP positive cells and estimate the titer. Infections were carried out in the presence of 8 μg/mL of polybrene and 10 mM HEPES. Virus-containing supernatant was removed after 24 hours and replaced with complete culture media. After 3 to 5 days, the transduced cells were analysed and isolated by flow cytometry which was carried out using FACScalibur and Cellquest software.

### 3.6. Immunoblot Analysis and Immunofluorescence Assay

RSCs were fixed, washed once in cold PBS, scraped, lysed with extraction buffer, and centrifuged at 10,000 rpm (9,730 g) for 10 minutes to remove insoluble material. Protein concentrations were determined using a protein assay kit (Bio-Rad, Hercules, CA, USA). Cell extracts in sample buffer were placed in boiling water for 5 minutes and then separated by 10% SDS-PAGE gel. After electrophoresis, the gel was transferred onto a PVDF membrane for immunoblotting. The membrane was blocked by incubation in non-fat milk at room temperature for 2 hours and incubated with SirT1 antibody (1:1000; Santa Cruz Biotechnology, Santa Cruz, CA, USA) for 2 hours at room temperature, washed five times with tris-buffered saline tween-20 (TBST), and incubated at room temperature with horseradish peroxidase-conjugated secondary antibody for 2 hours. The membrane was washed six times with TBST, and specific bands were made visible by chemiluminescence (ECL, Santa Cruz). For immunofluorescence study with GFAP, Thy-1, and opsin, the differentiated cells immunostained with monoclonal antibodies against GFAP (astoglial marker; 1:500; DAKO), Thy-1 (retinal ganglion cell marker;1:500; Chemicon), and rhodopsin (photoreceptor marker; 1:500; Chemicon) were diluted in PBS/3% Triton X-100/10% normal goat serum (NGS) and individually incubated with the coverslips for 2 hours at 37 °C. Coverslips were washed three times (10 minutes each) in PBS and incubated in appropriate secondary antibodies (1:200; Sigma, St Louis, MO, USA) for 30 minutes at 37 °C. Coverslips were rinsed three times in PBS and one time in distilled water and mounted on glass slides with Fluoesave (Calbiochem, La Jolla, CA, USA). The number of GFAP-, Thy-1 and opsin-positive cells were assessed in 10 non-overlapping fields for each sphere. The total number of cells in each field was determined by counterstaining cell nuclei with 4,6-diamidine-2-phenylindole dihydrochloride (DAPI; 1 mg/mL).

### 3.7. H_2_O_2_ Treatment

Cultured RSCs were washed with PBS twice and treated with 100 μM H_2_O_2_ in the culture medium. After 8 hours, the medium was removed, and the cells were collected for the subsequent RNA extraction.

### 3.8. Measurement of Intracellular ROS Production

The measurement of intracellular ROS production by the probe 2′,7′-dichlorofluorescein diacetate (DCFH-DA; Molecular Probes, Eugene, OR, USA) was mentioned previously [46]. In brief, cells were incubated with 5 μmol·L-1 DCFH-DA in culture medium for 30 minutes at 37 °C, DCFH-DA was metabolized by non-specific esterases to the non-fluorescence product, 2′,7′-dichlorofluoresceine, which was oxidized to the fluorescent product, DCF, by ROS. Then the cells were washed twice in PBS, trypsinized, resuspended in PBS and measured for their ROS content by flow cytometric analysis.

### 3.9. Statistical Analysis

The results are expressed as means ± SD. Statistical analyses were performed using *t*-test for comparing 2 groups, and one-way or two-way ANOVA, followed by Tukey’s test were used to detect the difference among 3 or more groups. A *P*-value is less than 0.05 considered statistically significant.

## 4. Conclusions

In conclusion, the level of SirT1 could efficiently monitor self-renewal and aging process in RSCs. RV could prevent that ROS-induced damage via increased retinal SirT1 expression.

## Figures and Tables

**Table 1.** Sequence of primer pairs used in real-time quantitative RT-PCR.

***Gene***	**Primer sequence (5′-3′)**
*Nestin*	F: GGGCCAGCACTCTTAGCTTTGATA
	R: TGAGCCTTCAGGGTGATCCAG
*Hes1*	F: CCAATTTGCCTTTCTCATCC
	R: GGAAGGTGACACTGCGTTAG
*Notch1*	F: CTCCAACTGTGACACCAACC
	R: GCACCCAGATCACACTCATC
*Pax6*	F: CAGCTTCACCATGGCAAACAAC
	R: AGGTATCATAACTCCGCCCATTCA
*Nrl*	F: ACGACCTGGGCAGTAGTCTCAA
	R: GTGTCGGAAGTCATCCAGTTCAA
*Brn3b*	F: CGATGCGGAGAGCTTGTCTTC
	R: GATGGTGGTGGTGGCTCTTACTCT
*GFAP*	F: ACCAGCTTACGGCCAACAGTG
	R: TGTCTATACGCAGCCAGGTTGTTC
*GAPDH*	F: CCTGCGACTTCAACAGCAACTC
	R: GTTGCTGTAGCCGTATTCATTGTCA

**Table 2.** Characteristics of patients for ocular retinal stem cell analysis.

Eye No.	Case No.	Age (yrs)	Sex	Eye	Death Cause	SirT1 activity
1	1	6	Male	Right	Traffic accident	+++++
2				Left		+++++
3	2	9	Male	Right	Traffic accident	+++++
4				Left		+++++
5	3	23	Male	Right	Traffic accident	+++
6				Left		++
7	4	28	Male	Right	Traffic accident	+++
8				Left		+++
9	5	33	Male	Right	Traffic accident	+++
10				left		++
11	6	41	Male	Right	Traffic accident	+++
12				Left		++
13	7	52	Male	Right	Stroke	+
14	8	61	Male	Right	Lung malignancy	++
15				Left		++
16	9	63	Female	Right	Traffic accident	+
17				Left		++
18	10	66	Male	Right	Traffic accident	+
19				Left		+
20	11	68	Male	Right	Stroke	+
21				Left		+
22	12	71	Male	Right	Colon malignancy	+
23				Left		+
